# Knowledge and Attitudes Related to Child Abuse and Neglect Among High School Students in the United States: A Multicenter Questionnaire Study

**DOI:** 10.7759/cureus.114144

**Published:** 2026-08-07

**Authors:** Venkata Ratna Kumar Rudravaram, Anuja Singaraju, Avika Gupta, Manasa Chinthala, Deepthi Maya, Keerthi Nalluri, Lavanya Karrotu, Srivani Swarna

**Affiliations:** 1 Dentistry, University of Washington School of Dentistry, Seattle, USA; 2 Dentistry, Loma Linda University School of Dentistry, Loma Linda, USA; 3 Biology, Lakeside School, Seattle, USA; 4 Paediatric and Preventive Dentistry, St. Joseph Dental College Eluru, Eluru, IND; 5 Dentistry, Central Ozarks Medical Centre, Richland, USA; 6 Dentistry, University of the Pacific, Arthur A. Dugoni School of Dentistry, San Francisco, USA; 7 Dentistry, Temple University Maurice H. Kornberg School of Dentistry, Philadelphia, USA; 8 Dentistry, Hermitage Family and Cosmetic Dentistry, Nashville, USA

**Keywords:** adolescents, attitudes, child abuse and neglect, knowledge, questionnaire development

## Abstract

Introduction

Child abuse and neglect (CAN) constitute a pervasive public health crisis affecting millions of children annually. Adolescents occupy a strategically important position in prevention efforts. However, population-level data on United States (U.S.) high school students' CAN literacy remain limited, and few psychometrically validated instruments exist for this population. This study aimed to (1) develop and validate a standardized 10-item questionnaire on the knowledge and attitudes toward child abuse and neglect (KA-CAN) and (2) characterize CAN knowledge and attitudes among a geographically diverse U.S. high school sample and to identify sociodemographic predictors.

Materials and methods

A cross-sectional survey was conducted across five U.S. geographic regions (February-April 2026), enrolling 478 students in grades 9-12 (mean age 16.1 ± 1.2 years). The KA-CAN instrument was developed through a five-phase process encompassing literature review, expert panel validation (Scale-Level Content Validity Index (S-CVI) = 0.91), focus group discussion, cognitive pretesting, and psychometric assessment. Descriptive statistics, bivariate comparisons, and multivariable linear regression were applied to analyze the data.

Results

The overall knowledge correct-response rate was 72.9% (2,090/2,868 item-responses), with mean knowledge and attitude scores of 4.37 ± 1.21 and 15.49 ± 3.62, respectively. Knowledge deficits included recognizing that abuse extends beyond physical harm (Item 1: 51.9%; 248/478) and knowledge of mandatory reporting obligations (Item 5: 60.5%; 289/478). Prior CAN education was the strongest predictor of both knowledge (B = 0.62, p < 0.001) and attitudes (B = 1.42, p < 0.001). The KA-CAN demonstrated good internal consistency (Cronbach's α = 0.81) and test-retest reliability (intraclass correlation coefficient (ICC) = 0.83).

Conclusion

Substantial gaps in CAN knowledge and reporting comfort persist among U.S. adolescents. The validated KA-CAN instrument provides a reliable standardized tool for future evaluation, and findings support the universal integration of evidence-based CAN curricula into U.S. high school programs.

## Introduction

Child abuse and neglect (CAN) encompasses all forms of physical and emotional ill-treatment, sexual abuse, neglect, negligent treatment, and commercial or other exploitation that result in actual or potential harm to a child’s health, survival, development, or dignity, as defined by the World Health Organization [1]. CAN affects an estimated one billion children aged two to 17 years annually worldwide [2]. In the United States, child protective services received approximately 3.0 million referrals involving 5.4 million children in 2021, representing a victimization rate of 8.1 per 1,000 children [3]. Children experiencing maltreatment are at significantly increased risk for a wide range of adverse outcomes across the life course, including post-traumatic stress disorder (PTSD), depression, anxiety disorders, substance use disorders, and suicidal ideation [4,5]. Beyond mental health sequelae, CAN has been associated with substantially increased risks for cardiovascular disease, obesity, diabetes, and early mortality in adulthood, underscoring the importance of early maltreatment as a social determinant of health [6,7].

Prevention and early detection are the most cost-effective measures to reduce the burden of CAN [8]. High school students possess the cognitive maturity to understand complex social and legal concepts, the social reach to witness or learn of abuse in their peer networks, and the moral reasoning skills to make independent decisions about reporting [9]. Adolescents are often witnesses or informal confidants in CAN situations, positioning them in a vital role for early identification and help-seeking [10].

School-based CAN programs aimed at improving adolescent knowledge and attitudes have shown substantial progress in disclosure rates and protective peer norms [11,12]. However, population-level data describing CAN knowledge and attitudes among U.S. high school students remain limited. Existing research largely focuses on college students, healthcare trainees, or child-serving professionals [13], rendering high school populations - a developmentally unique cohort bridging childhood and early adulthood - severely underrepresented. Studies focused on adolescents are often constrained by small convenience samples, single-site designs, and unvalidated instruments, resulting in a significant evidence gap [14,15]. Systematic reviews of CAN assessment instruments have repeatedly identified the absence of psychometrically valid questionnaires for non-clinical, school-based adolescent samples [15,16].

Against this backdrop, the present study had two primary aims: (1) to develop and validate a standardized 10-item questionnaire on knowledge and attitudes toward child abuse and neglect (KA-CAN) through a five-phase development process; and (2) to characterize CAN knowledge and attitudes among a geographically diverse U.S. high school sample and to identify sociodemographic predictors. To address these aims, the following research questions (RQ) were formulated: (RQ1) What is the level of CAN knowledge among U.S. high school students in grades 9-12, as measured by the KA-CAN instrument, and which specific knowledge domains show the greatest deficits? (RQ2) What are the prevailing attitudes toward CAN among U.S. high school students? (RQ3) Does prior formal education in CAN significantly and independently influence both CAN knowledge and attitudes, after controlling for other sociodemographic variables? (RQ4) Do CAN knowledge and attitudes differ significantly across sociodemographic subgroups, including gender, grade level, school type, and geographic region?

## Materials and methods

Study design and setting

A cross-sectional, questionnaire-based study was conducted across 20 high schools that participated across the five regions: four schools in the Northeast (Massachusetts and New York), four in the Southeast (Florida and Georgia), four in the Midwest (Illinois and Ohio), four in the Southwest (Texas and Arizona), and four in the West (California and Washington). Of the 20 schools, 11 were public, and nine were private. Participant numbers per region ranged from 88 to 102, spanning five U.S. geographic regions from February to April 2026. The study was designed and reported in accordance with the Strengthening the Reporting of Observational Studies in Epidemiology (STROBE) statement [17].

Study population

Eligible participants were full-time students enrolled in public or private U.S. high schools, aged 14 to 18 years, currently attending grades 9 through 12, capable of responding to English-language items, and willing to provide voluntary assent. Students were excluded if they or their parents declined consent, if they had severe or profound intellectual disabilities as documented in their Individual Education Plan (IEP) that would preclude independent reading at a grade 6 reading level, if they returned incomplete questionnaires (defined as three or more of 10 items left blank), or if they had participated in a prior pilot or pretesting phase.

Sample size

The required sample size was estimated using the single-proportion formula: $n=\frac{Z_{\alpha/2}^{2}\times P(1-P)}{d^{2}}$ where *n* denotes the required sample size, $Z_{\alpha/2}$ is the standard normal deviate for the desired confidence level, *P* is the anticipated proportion, and *d* is the acceptable margin of error. Based on a 95% confidence level ($Z_{\alpha/2}=1.96$), an anticipated proportion of 50% (*P=0.50*), and a margin of error of 5% (*d=0.05*), the minimum required sample size was estimated to be 385 participants. After accounting for an anticipated non-response rate of 15%, the adjusted sample size was 453 participants. To ensure adequate geographic and grade-level representation, the target sample size was increased to 500 participants. The sample size calculation was independently verified using G*Power software (Version 3.1.9.7; Heinrich Heine University, Düsseldorf, Germany).

Sampling strategy

A multistage, mixed purposive-random sampling approach was employed. Schools were purposively selected for regional diversity; within each school, classrooms were randomly selected by lottery; all eligible students in selected classrooms were invited to participate, constituting whole-classroom enrollment within the systematically selected units. School principals in each region were contacted by email and telephone; 27 schools were approached in total, and 20 agreed to participate (seven declined, citing scheduling constraints or administrative burden; refusal rate 26%). Within each consenting school, the school administrator provided a complete list of all active classrooms for grades 9-12. Two classrooms per grade (i.e., eight classrooms per school) were randomly selected by lottery, and all eligible students present on the day of administration were invited. Mean class size was 26.2 students (range: 18-31), yielding approximately 50 students invited per school.

Questionnaire development

The questionnaire was constructed through a structured five-phase development process. A systematic literature search of PubMed and Scopus was performed using the terms "child abuse," "child neglect," "CAN knowledge," "CAN attitudes," and "adolescent," and identified 47 studies informing construct identification. Fourteen experts were invited for content validity review; 10 responded (response rate 71.4%), representing child psychology, public health, paediatric dentistry, paediatrics, social work, and education, each with a minimum of five years' child protection experience.

Phase 1: Establishing the Conceptual Framework

A comprehensive review of the CAN literature identified six core constructs: (1) ability to differentiate and define physical, emotional, and sexual abuse and neglect; (2) knowledge of CAN prevalence and conditions that increase its probability; (3) knowledge of legal mandatory reporting obligations; (4) understanding of the immediate and long-term consequences of abuse on victims; (5) attitudes toward victims, perpetrators, and the role of society in prevention; and (6) perceived self-efficacy and willingness to report suspected abuse.

Phase 2: Item Generation

A multidisciplinary team with expertise in child psychology, public health, pediatric nursing, social work, and education generated an initial bank of 45 candidate items across three formats: dichotomous yes/no questions, multiple-choice items, and five-point Likert-scale statements (1 = “Strongly Disagree” to 5 = “Strongly Agree”). Independent review followed by group consensus reduced the pool to 28 items meeting pre-established criteria for conceptual alignment, age-appropriate language (approximately grade 8 reading level), and absence of leading or double-barreled phrasing.

Phase 3: Focus Group Discussion

Three focus group sessions were held with high school students, class teachers, and child protection practitioners (eight to 10 participants each). A semi-structured guide covering item clarity, cultural appropriateness, age-appropriateness, and construct relevance was used. Transcripts were analyzed using a six-phase thematic analysis framework by two independent coders (Cohen's kappa = 0.82). Revisions resulted in the removal of four items, rephrasing of several items, merging of three pairs of overlapping items, and addition of two new items, yielding a refined pool of 23 items.

Phase 4: Cognitive Pretesting

The 23-item version was piloted with 30 high school students. Items were flagged for deletion or consolidation if the missing-response rate exceeded 10%, response variance fell in the lowest quartile, or mean inter-item correlation within the assigned construct domain was below 0.20. Of 13 items removed, eight were deleted due to high missingness or minimal variance, and five were merged to eliminate redundancy. This yielded a 10-item instrument for the validation phase.

Phase 5: Validity Assessment

Content validity was evaluated by a panel of 10 subject-matter experts using a four-point scale rating each item on relevance, clarity, simplicity, and ambiguity. Item-Level Content Validity Index (I-CVI) scores were calculated; items falling below 0.78 were revised or removed. The overall Scale-Level Content Validity Index (S-CVI)/Average for the final 10-item instrument was 0.91. Face validity was confirmed by 15 independent students and five teachers, all of whom rated the items as clear, relevant, and age-appropriate. For the attitude subscale (Items 7-10), an exploratory factor analysis (EFA) (principal axis factoring, oblimin rotation) yielded a single-factor solution accounting for 61.4% of variance with all loadings ≥ 0.52, supporting unidimensionality. For the knowledge subscale (Items 1-6), Rasch analysis confirmed a unidimensional structure, with information-weighted mean square (INFIT MnSq) values ranging from 0.84 to 1.18. Known-groups validity was supported by significantly higher scores in students with prior CAN education versus those without (both p < 0.001). Test-retest reliability was assessed in a subsample of 42 students over a 14-day interval using a two-way mixed-effects intraclass correlation coefficient (ICC) model for absolute agreement (ICC_2,1_): knowledge ICC = 0.83 (95% CI: 0.72-0.90); attitude ICC = 0.79 (95% CI: 0.66-0.87).

Test-Retest Reliability

This was assessed in a convenience subsample of 42 high school students drawn from one of the five participating regions who were not included in the main study sample. Participants completed the KA-CAN on two occasions separated by a 14-day interval, a duration selected to minimize both recall effects and genuine change in CAN knowledge or attitudes. Administration conditions were identical to the main study. The ICC was computed using a two-way mixed-effects model for absolute agreement. The ICC for total knowledge score was 0.83 (95% CI: 0.72-0.90) and for total attitude score was 0.79 (95% CI: 0.66-0.87), both indicating acceptable test-retest stability.

Final questionnaire: the KA-CAN 10-item instrument

The final 10-item KA-CAN questionnaire (Table 1) comprises six knowledge items and four attitude items. Each knowledge item is answered as True/False/I Don’t Know, and each attitude item is rated on a five-point Likert scale (1 = Strongly Disagree to 5 = Strongly Agree).

**Table 1:** The Knowledge and Attitudes Toward Child Abuse and Neglect (KA-CAN) Questionnaire

No.	Question Item	Response Format/Domain
Section A: Knowledge Items (Items 1-6)		
1	Child abuse refers only to physical harm caused to a child by a caregiver or adult.	True/False/I Don’t Know
2	Emotional and psychological maltreatment (such as constant criticism, humiliation, and rejection) is recognized as a form of child abuse.	True/False/I Don’t Know
3	Child neglect occurs when a parent or caregiver fails to provide a child with basic necessities such as food, shelter, clothing, medical care, or supervision.	True/False/I Don’t Know
4	Children from families experiencing poverty, domestic violence, or parental substance abuse are at higher risk of experiencing abuse and neglect.	True/False/I Don’t Know
5	In most United States, certain professionals who work with children including teachers, nurses, doctors are legally required to report suspected child abuse to authorities.	True/False/I Don’t Know
6	Children who experience abuse or neglect may suffer long-term effects such as depression, post-traumatic stress disorder (PTSD), learning difficulties, and problems with relationships.	True/False/I Don’t Know
Section B: Attitude Items (Items 7-10)		
7	Children who are abused or neglected are not responsible for what happens to them; the responsibility lies entirely with the abusive adult.	5-Point Likert Scale (1=Strongly Disagree - 5=Strongly Agree) Domain: Victim Attribution
8	I believe that child abuse and neglect is a serious social problem that requires community-wide attention and action, not just intervention by families.	5-Point Likert Scale (1=Strongly Disagree - 5=Strongly Agree) Domain: Societal Responsibility
9	If I suspected that a classmate or child I knew was being abused or neglected, I would feel comfortable reporting it to a trusted adult or authority.	5-Point Likert Scale (1=Strongly Disagree - 5=Strongly Agree) Domain: Help-Seeking Behavior
10	Schools should include education about child abuse, neglect, and personal safety as a regular part of the curriculum for all students.	5-Point Likert Scale (1=Strongly Disagree - 5=Strongly Agree) Domain: Preventive Attitudes

Data collection and ethics

Active written parental consent was obtained at least two weeks prior to data collection; student assent was obtained in writing on the day of administration. Students were informed that participation was voluntary, responses were anonymous, any item could be skipped, and withdrawal was permitted at any time without penalty. All research assistants completed state-specific mandatory reporting training; students were informed that confidentiality limits applied if responses indicated current abuse or imminent harm, consistent with applicable state law. Questionnaires were completed on paper; teachers remained outside the classroom during administration to minimize social desirability bias. Following institutional ethics approval (IRB/Ethics Reference: IEC/NDCH/2020/P-41/A) and school authorization, trained research assistants administered the questionnaire in classroom settings. Anonymity was ensured through submission in sealed envelopes; no identifiable information was recorded. The study complied with the Declaration of Helsinki and 45 CFR Part 46. A psychosocial resource list (including the Childhelp National Child Abuse Hotline: 1-800-422-4453) was provided at completion.

Statistical analysis

Data were entered into Microsoft Excel 2016 (Microsoft Corp., Redmond, WA, USA). Analyses were conducted using IBM SPSS Statistics for Windows, Version 27 (Released 2019; IBM Corp., Armonk, New York, United States) and R v4.3.0 (R Foundation for Statistical Computing, Vienna, Austria). Descriptive statistics summarized demographic and questionnaire variables. For knowledge items, "I Don't Know" and blank responses were both scored as 0 (incorrect), and correct responses scored as 1, yielding a total knowledge score ranging from 0 to 6; attitude items were summed to yield a total attitude score ranging from 4 to 20. Attitude scores were classified a priori as High (≥ 16), Moderate (12-15), or Low (≤ 11). Score normality was assessed via the Shapiro-Wilk test. Bivariate comparisons used independent-samples t-tests, one-way analysis of variance (ANOVA), or nonparametric equivalents as appropriate. Multivariable linear regression identified independent predictors of knowledge and attitude scores. Statistical significance was set at p < 0.05 (two-tailed).

## Results

Sample characteristics

Of 500 invited students, 478 were included in the final analysis (22 excluded: 14 for incomplete responses, eight for prior pretesting participation), yielding a response rate of 95.6%. Item-level missingness ranged from 0.4% to 2.1%, with no item exceeding 3% missing; complete-case analysis was used. Sociodemographic characteristics are summarized in Table 2. Mean age was 16.1 ± 1.2 years; grade-level representation was approximately equal across grades 9 through 12. Females constituted 52.7% of the sample and 39.1% reported prior formal CAN education. Public school students comprised 55.9% (n = 267) and private school students 44.1% (n = 211) of the sample.

**Table 2:** Sociodemographic Profile of Participants (N = 478) CAN: child abuse and neglect

Variable	n	%
Age (mean ± SD): 16.1 ± 1.2 years; range 14-18 years		
Grade Level		
Grade 9	118	24.7
Grade 10	124	25.9
Grade 11	122	25.5
Grade 12	114	23.9
Gender		
Female	252	52.7
Male	214	44.8
Non-binary/Other	12	2.5
School Type		
Public	267	55.9
Private	211	44.1
Prior CAN Education		
Yes	187	39.1
No	291	60.9
Total (final sample)	478	95.6% response rate

CAN knowledge

The overall correct-response rate across the six knowledge items was 72.9% (2,090/2,868 total responses). Item-level rates ranged from 51.9% (248/478) (Item 1: definitional scope of abuse) to 88.1% (421/478) (Item 6: long-term consequences). Item 5, addressing mandatory reporting obligations, achieved a correct-response rate of only 60.5% (289/478), with 23.4% (112/478) providing incorrect responses and 16.1% (77/478) selecting “Don’t Know.” Item-level distributions are detailed in Table 3.

**Table 3:** Knowledge Item Response Distribution (N = 478) CAN: child abuse and neglect; DV: domestic violence

Item	Description	Correct, n (%)	Incorrect, n (%)	Don't Know, n (%)	Correct Response
1	Scope of child abuse beyond physical harm	248 (51.9)	139 (29.1)	91 (19.0)	False
2	Emotional/psychological maltreatment as abuse	352 (73.6)	68 (14.2)	58 (12.1)	True
3	Definition of child neglect	412 (86.2)	38 (7.9)	28 (5.9)	True
4	Risk factors for CAN (poverty, DV, substance use)	368 (77.0)	62 (13.0)	48 (10.0)	True
5	Mandatory reporting obligations for professionals	289 (60.5)	112 (23.4)	77 (16.1)	True
6	Long-term psychosocial consequences of CAN	421 (88.1)	31 (6.5)	26 (5.4)	True
Overall correct response rate: 2,090/2,868 (72.9%)					

The mean total knowledge score was 4.37 ± 1.21 out of six. Score classification revealed high knowledge (scores of 5-6) in 56.9% (272/478) of students, moderate knowledge (scores of 3-4) in 35.1% (168/478), and low knowledge (scores of 0-2) in 7.9% (38/478). The distribution was slightly negatively skewed (skewness = -0.82).

CAN attitudes

Attitude scores were broadly favorable across all four items (Table 4). Item 10 (curriculum integration) received the highest mean score (4.08 ± 1.10; 78.7% (376/478) Agree/Strongly Agree). Item 9 (comfort with reporting) had the lowest mean score (3.50 ± 1.22); 23.0% (110/478) of respondents selected Disagree or Strongly Disagree, indicating residual hesitancy toward reporting. Mean total attitude score was 15.49 ± 3.62 out of 20. Attitude score classification yielded: High (≥ 16): 67.4% (n = 322); Moderate (12-15): 24.7% (n = 118); Low (≤ 11): 7.9% (n = 38).

**Table 4:** Attitude Item Response Distribution (N = 478) SA: Strongly Agree; A: Agree; N: Neutral; D/SD: Disagree or Strongly Disagree

Item	Domain	SA, n (%)	A, n (%)	N, n (%)	D/SD, n (%)	Mean ± SD
7	Victim Attribution	189 (39.5)	161 (33.7)	78 (16.3)	50 (10.5)	3.87 ± 1.12
8	Societal Responsibility	196 (41.0)	172 (36.0)	64 (13.4)	46 (9.6)	4.04 ± 1.08
9	Help-Seeking/Reporting Comfort	127 (26.6)	175 (36.6)	66 (13.8)	110 (23.0)	3.50 ± 1.22
10	Preventive/Curriculum Attitudes	218 (45.6)	158 (33.1)	54 (11.3)	48 (10.0)	4.08 ± 1.10
Mean total attitude score: 15.49 ± 3.62						

Bivariate and regression analyses

Bivariate comparisons revealed statistically significant differences across multiple sociodemographic subgroups (Table 5). Female students outperformed male students on both knowledge (4.52 ± 1.14 vs. 4.18 ± 1.28; p = 0.018) and attitude scores (16.12 ± 3.42 vs. 14.72 ± 3.74; p = 0.001). A consistent positive gradient was observed across grade levels for both scores (both p ≤ 0.012); post-hoc Tukey's Honestly Significant Difference (HSD) confirmed significant pairwise differences between grades 9 and 12 (knowledge: p < 0.001; attitudes: p = 0.008). Private school students scored modestly but significantly higher on both scales (knowledge: p = 0.012; attitudes: p = 0.003). No significant regional differences were detected (all p > 0.40).

**Table 5:** Bivariate Comparisons of KA-CAN Scores by Sociodemographic Subgroup * denotes statistically significant p-values (p < 0.05); ** denotes highly significant p-values (p < 0.001). Gender comparisons: independent-samples t-test; Grade level comparisons: one-way ANOVA with post-hoc Tukey HSD; School type comparisons: independent-samples t-test; Prior CAN education comparisons: independent-samples t-test. Regional comparisons not shown (all p > 0.40). Post-hoc Tukey HSD confirmed significant pairwise differences between grades 9 and 12 (knowledge: p < 0.001; attitudes: p = 0.008). CAN: child abuse and neglect; KA-CAN: knowledge and attitudes toward child abuse and neglect; ANOVA: analysis of variance; HSD: Honestly Significant Difference

Subgroup	Knowledge, Mean ± SD	Attitude, Mean ± SD	P-value (Knowledge)	Test Statistic	P-value (Attitude)
Gender					
Female	4.52 ± 1.14	16.12 ± 3.42	0.018*	t = 2.37/t = 3.37	0.001**
Male	4.18 ± 1.28	14.72 ± 3.74			
Grade Level					
Grade 9	3.98 ± 1.32	14.82 ± 3.88	<0.001**	F = 8.14/F = 3.82	0.012*
Grade 10	4.22 ± 1.18	15.28 ± 3.61			
Grade 11	4.48 ± 1.12	15.72 ± 3.42			
Grade 12	4.72 ± 1.08	16.12 ± 3.28			
School Type					
Private	4.62 ± 1.08	16.34 ± 3.28	0.012*	t = 2.52/t = 2.98	0.003**
Public	4.28 ± 1.26	15.12 ± 3.72			
Prior CAN Education					
Yes	4.82 ± 1.02	16.42 ± 3.28	<0.001**	t = 5.71/t = 3.83	<0.001**
No	4.08 ± 1.24	14.88 ± 3.72			

In multivariable linear regression, prior formal CAN education was the strongest and most consistent predictor of both knowledge (β = 0.62, p < 0.001) and attitude scores (β = 1.42, p < 0.001), followed by grade level (knowledge: β = 0.28, p < 0.001; attitudes: β = 0.42, p = 0.001) and female gender (knowledge: β = 0.34, p = 0.018; attitudes: β = 1.40, p = 0.001).

## Discussion

This cross-sectional study provides a comprehensive characterization of CAN knowledge and attitudes among U.S. high school students across five geographic regions, using the newly validated KA-CAN instrument. Most participants demonstrated high knowledge (56.9%; 272/478) and favorable attitudes (67.4%; 322/478), but clinically meaningful gaps remain - particularly in recognizing the full spectrum of abuse types, knowledge of mandatory reporting, and willingness to report - with clear implications for prevention programming. The KA-CAN demonstrated preliminary content and structural validity (S-CVI = 0.91; single-factor EFA for attitudes; Rasch analysis confirming unidimensionality of the knowledge subscale), acceptable internal consistency (Kuder-Richardson Formula 20 (KR-20) = 0.76 for knowledge; α = 0.74 for attitudes), and satisfactory test-retest reliability (knowledge ICC = 0.83; attitude ICC = 0.79), supporting its preliminary use in research and program evaluation contexts pending further external validation.

The overall correct-response rate (72.9%) is similar to studies conducted among adolescents and young adults in high-income countries, which primarily report rates between 60% and 85% [13]. The lowest rate (Item 1: 51.9%; 248/478), which measured recognition that abuse involves more than physical harm, demonstrates a well-documented pattern of under-recognition of emotional and psychological abuse. A high-quality survey of U.S. adults found that only 40% would be likely to report emotional abuse to authorities, compared with 95% for sexual abuse and 84% for physical abuse [1]. These findings indicate that educational interventions should address the entire spectrum of maltreatment types, rather than defaulting to the physical harm paradigm.

The relatively low correct-response rate for mandatory reporting obligations (Item 5: 60.5%; 289/478) is of concern because adolescents’ awareness of the legal reporting system affects their expectations of trusted adults and their own help-seeking behavior [18]. Children and adolescents are more likely to disclose abuse when they perceive trusted adults as approachable and capable of responding protectively [19]. Reinforcing the teaching of reporting systems in school curricula is therefore a clear programmatic objective [20].

The highest rates of correct responses were for Item 3 (definition of neglect, 86.2%; 412/478) and Item 6 (long-term consequences, 88.1%; 421/478), suggesting that awareness of neglect and the psychosocial consequences of CAN may be effectively communicated through existing informal socialization channels, including media, family conversations, and general health education, consistent with prior literature [21]. Items 3 and 6 also showed near-ceiling effects, which limits their discriminatory power and is acknowledged as a limitation of the current instrument that future iterations should address.

The strong support for curriculum integration (Item 10; 78.7% (376/478) Agree/Strongly Agree) is consistent with recommendations from the Centers for Disease Control and Prevention (CDC) Violence Prevention Technical Package [22]. However, the finding that approximately one in four students felt uncomfortable reporting suspected abuse (Item 9; 23.0% (110/478) Disagree/Strongly Disagree) is of particular importance to program design. This aligns with a substantial body of literature describing adolescent reporting barriers such as fear of retaliation, uncertainty about institutional responses, loyalty conflicts, and normalization of abusive behaviors [23,24]. A recent qualitative meta-analysis highlighted self-efficacy barriers as particularly relevant for mid-to-late adolescence [25]. These data demonstrate the necessity of integrating social-emotional learning components into knowledge-based curricula to enhance reporting self-efficacy, normalize help-seeking, and offer concrete disclosure protocols [26].

Prior formal CAN education was the strongest modifiable predictor of knowledge and attitudes (p < 0.001), independently of demographic factors. This supports evidence from established school-based programs, including Darkness to Light’s Stewards of Children, Speak Up Be Safe, and Good Touch/Bad Touch, emphasizing the modifiability of CAN literacy through intentional teaching [27,11]. However, prior CAN education was measured via a single unverified self-report item, which is susceptible to recall bias and inconsistent respondent definitions of "formal" education; the nature, content, and duration of reported prior education were not assessed, precluding dose-response conclusions.

Female students scored higher on both knowledge and attitudes. One plausible hypothesis is that gendered socialization patterns may promote greater empathy or engagement with socially sensitive content among female adolescents; however, these constructs were not measured in this study, and this interpretation remains speculative. Alternative explanations include differential prior exposure, gender-related differences in social desirability responding, or unmeasured school-level factors [28]. These results support gender-responsive educational initiatives, including explicit attention to masculinity norms that may impede disclosure and the inclusion of male-inclusive messaging, in accordance with WHO INSPIRE strategies [29].

The absence of significant geographic differences across five U.S. regions (all p > 0.40) may reflect the nationally homogenizing effects of social media, federal awareness initiatives, and the increasingly pervasive integration of CAN curriculum components. This finding contradicts hypotheses regarding regional heterogeneity driven by state-level variations in child protective services capacity and mandatory reporting laws [30]. Future research could identify more subtle differences through granular rural/urban/suburban stratification.

The five-phase development process, encompassing expert content validation (S-CVI = 0.91), focus group input from the target population, and iterative pretesting, represents a methodological standard rarely achieved by instruments used in comparable CAN research [15]. Initial psychometric evidence supports the preliminary utility of the KA-CAN for research and program evaluation; however, further structural, convergent, and criterion validity assessment in independent samples remains necessary before the instrument can be considered fully validated for clinical or population-surveillance use.

Strengths of this study include a substantial, geographically diversified sample, a comprehensively validated instrument, adherence to STROBE reporting guidelines, and concurrent evaluation of knowledge, attitudes, and sociodemographic variables. Several significant limitations of this study warrant acknowledgement. The cross-sectional design precludes causal inference, and the association between CAN education and outcomes may be partially attributable to self-selection. Self-reported data may be influenced by social desirability bias. The purposive selection of schools, absence of socioeconomic, racial/ethnic, and victimization-history data, and English-only administration each limit generalizability. Incomplete psychometric validation, particularly the absence of CFA, measurement invariance testing, and criterion validity data, should currently be regarded as an instrument with preliminary rather than comprehensive validity evidence. School-type differences should be interpreted with caution in the absence of socioeconomic controls. Future studies should consider cross-national validation and cultural adaptation of the KA-CAN instrument.

## Conclusions

This study demonstrates that, while U.S. high school students generally possess foundational CAN knowledge and favorable preventive attitudes, significant deficits still remain, particularly regarding the full spectrum of abuse types, mandatory reporting obligations, and self-efficacy for reporting. Prior formal education in CAN, higher grade level, female gender, and enrollment in a private school each independently associated with higher CAN literacy. The validated KA-CAN questionnaire offers a brief, reliable, and reproducible tool for future research and program evaluation use. These findings establish a meaningful association between prior formal CAN education and higher CAN literacy, providing a rationale for prioritizing well-designed controlled trials evaluating structured, theory-grounded CAN curricula in U.S. high school settings. Use of the KA-CAN for population surveillance would require additional evidence regarding measurement invariance, sensitivity to change, and external validation in independent samples.
